# Somatic mutations in the mitochondria of rheumatoid arthritis synoviocytes

**DOI:** 10.1186/ar1752

**Published:** 2005-04-28

**Authors:** Tanya R Da Sylva, Alison Connor, Yvonne Mburu, Edward Keystone, Gillian E Wu

**Affiliations:** 1 Department of Biology, York University, Toronto, Ontario, Canada; 2 The Wellesley Toronto Arthritis and Immune Disorder Research Centre, University Health Network, Toronto, Ontario, Canada; 3 Department of Medicine, University of Toronto, Mount Sinai Hospital, Toronto, Ontario, Canada

## Abstract

Somatic mutations have a role in the pathogenesis of a number of diseases, particularly cancers. Here we present data supporting a role of mitochondrial somatic mutations in an autoimmune disease, rheumatoid arthritis (RA). RA is a complex, multifactorial disease with a number of predisposition traits, including major histocompatibility complex (MHC) type and early bacterial infection in the joint. Somatic mutations in mitochondrial peptides displayed by MHCs may be recognized as non-self, furthering the destructive immune infiltration of the RA joint. Because many bacterial proteins have mitochondrial homologues, the immune system may be primed against these altered peptides if they mimic bacterial homologues. In addition, somatic mutations may be influencing cellular function, aiding in the acquirement of transformed properties of RA synoviocytes. To test the hypothesis that mutations in mitochondrial DNA (mtDNA) are associated with RA, we focused on the *MT-ND1 *gene for mitochondrially encoded NADH dehydrogenase 1 (subunit one of complex I – NADH dehydrogenase) of synoviocyte mitochondria from RA patients, using tissue from osteoarthritis (OA) patients for controls. We identified the mutational burden and amino acid changes in potential epitope regions in the two patient groups. RA synoviocyte mtDNA had about twice the number of mutations as the OA group. Furthermore, some of these changes had resulted in potential non-self MHC peptide epitopes. These results provide evidence for a new role for somatic mutations in mtDNA in RA and predict a role in other diseases.

## Introduction

Rheumatoid arthritis (RA) is a chronic inflammatory autoimmune disease. It is multigenic, possibly triggered by exposure to viruses or bacteria, and, it is expected, other environmental stimuli. Consistent with this concept is the strong genetic association with the HLA-DR allele that contains a QK/RAA amino acid motif in its third hypervariable region, namely several alleles of the HLA-DRβ1 gene. The precise role of HLA-DR in pathogenesis is unknown, although its role in antigen presentation is the most obvious [1]. *In vitro *T-cell proliferation assays using the susceptible major histocompatibility complex (MHC) alleles has led to the discovery of a multiplicity of putative peptide autoantigens including collagen type II, cartilage link protein, heat shock proteins, and aggrecan [1].

There are nonimmune components to RA. RA synovial fibroblasts have many features of transformed cells – including the expression of oncogenes – and they have been shown to invade and destroy cartilage in the absence of T cells [2,3]. The acquisition of these transformed characteristics is thought to be aided by increased somatic mutations caused by reactive oxygen species (ROS) and reactive nitrogen species (RNS) produced endogenously within the inflamed joint [4]. Other studies linking ROS and RNS damage to decreased apoptosis have found ROS-associated damage to p53. The mutated p53 was a dominant negative, suggesting that p53 mutations help protect pathogenic cells from apoptosis [5-7].

Mitochondrial DNA (mtDNA) damage may complement damage to nuclear regulatory genes and have a causative role in the transformation of RA synovial cells. There is limited and sometimes contradictory evidence available concerning the ability of mtDNA mutations to lead to increased or decreased apoptosis [8]. Alterations of mtDNA are now being found in many tumor types and there is evidence that these mutations may contribute to the progression of human cancer [9,10].

There is growing evidence that somatic mutations within protein-coding genes of mtDNA may be recognized by the immune system: damaged mtDNA results in increased expression of MHC class I; and both MHC class I and class II can present mitochondrial peptides [11,12]. Mutated mitochondrial peptides in resident cells may, therefore, be aiding in the recruitment of immunological factors such as cytotoxic T cells to the RA joint.

Complex I – NADH (reduced nicotinamide-adenine dinucleotide) dehydrogenase is exceptionally susceptible to defects due to mtDNA mutations, because it has the most subunits encoded by mtDNA. Cells with complex I defects have also been shown to produce a higher amount of superoxide *in vivo *[8,13]. Therefore, defects in complex I may help perpetuate a vicious cycle of oxidative damage. The murine homologue of subunit 1 of complex I – NADH dehydrogenase (mtND1) plays a critical role in self recognition. The maternally transmitted antigen of rats and mice is the product of a class I molecule that presents the maternal transplantation factor derived from the amino terminus of mtND1 [14]. These findings provide evidence that antigenic peptides of human mtND1 may be displayed and recognized by the immune system.

To test the hypothesis that mutations in mitochondria play a role in RA, we examined the *MT-ND1 *gene of RA synoviocytes. As a control we chose synoviocytes from patients with osteoarthritis (OA). This disease was chosen because it is primarily a noninflammatory syndrome that is not thought to be directly dependent on the immune system. RA synoviocyte mtRNA had about twice the number of mutations as the OA group, revealing a greater mutational burden in RA. Furthermore, some of these changes resulted in changes that were potential non-self MHC peptide epitopes.

## Materials and methods

### RNA extraction from tissue and fibroblast lines

The protocol for the use of human tissues was approved by ethics review committees at the University Health Network and St Michael's Hospital, Toronto, Canada. Synovial tissues were obtained from RA and OA patients at the time of arthroplasty. The patients were not chosen by any criterion other than disease diagnosis. A portion of each sample was added to Trizol (Sigma Aldrich, St. Louis, MO, USA) and stored at -80°C until it was processed according to the manufacturer's instructions. Synovial fibroblast lines derived from the synovial tissue were established as previously described [15]. The fourth passage was used for all RA and OA lines. Cells were maintained in OptiMEM (Invitrogen Life Technologies, Carlsbad, CA, USA) supplemented with 10% fetal bovine serum and 1% antibiotic–antimycotic. They were cultured at 37°C in a humidified chamber containing 95% air, 5% CO_2_.

### RT-PCR and sequencing

Total RNA extracts from the fibroblasts and tissue of RA and OA patient samples were amplified using RT-PCR. This was a two- step protocol using the materials and methods included with the DuraScript RT-PCR Kit (Sigma Aldrich). In brief, first-strand cDNA was generated using 50 ng of total RNA, random nonamers for extension primers, and enhanced avian myeloblastosis virus (AMV) reverse transcriptase. Three PCR reactions were then performed using 5 μl first-strand cDNA in each 50 μl PCR. The primer pairs and amplification conditions are described in Table 1 and have been published previously [16].

Direct sequencing of PCR products does not detect low levels of heteroplasmy; therefore the PCR fragments were cloned into a TA vector (using protocols and materials provided in the TOPO TA Cloning^® ^Kit for Sequencing with One Shot^® ^TOP10 Chemically Competent *E. coli*; Invitrogen, Carlsbad, CA, USA). Approximately ten colonies from each patient sample were chosen and sequenced using T3 and T7 primers. To rule out sequencing errors, only areas of complete identity (between the T3 and T7 sequence) were aligned with the mitochondrial Anderson Reference Sequence [17]. Nucleotide changes from the reference sequence were recorded and then entered into the online program MitoAnalyzer (National Institute of Standards and Technology, Gaithersburg, MD, USA; ; 2000) which displays the sequence and any amino acid changes resulting and the position number affected.

The same amplification and sequencing procedure as above was followed using PCR primers (Table 1) and conditions previously published for a nuclear gene, that for dihydrolipamide dehydrogenase (*DLD*) [18,19]. Nucleotide changes from the NCBI (National Center for Biotechnology Information) reference sequence (gi:5016092) were recorded and corresponding amino acid changes determined.

To control for errors induced by PCR and cloning/transformation, three plasmids containing cloned fragments were amplified and sequenced as above (approximately 18,000 bp in both directions). A methodological error frequency was calculated (0.00095 errors/bp for total mutational burden and 0.00063 errors/bp for expressed mutational burden) and subtracted from the final mutational burden data before statistical analyses. Throughout this report, the data presented are corrected for methodological error.

### Mutational burden comparisons

**Figure 1:**
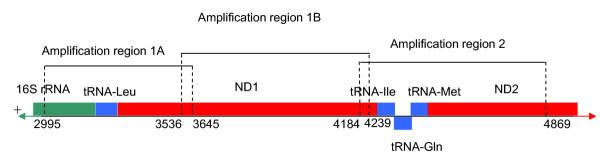
The three amplified and sequenced regions of mtDNA, corresponding to primers given in Table 1. tRNA-Gln is encoded on the negative (or light) strand of mtDNA. ND1, NADH-dehydrogenase subunit 1; ND2, NADH dehydrogenase subunit 2.

The mutational burden of OA and RA patients was defined as the number of mutations identified within that group divided by the total number of base pairs analyzed. This was then further separated into two measurements, total mutational burden (all mutations) and expressed mutational burden (the number of amino acid changes in the *MT-ND1 *cDNA from each amplified region; see Fig. 1). All patients sequenced with the first set of *MT-ND1 *primers (1A) had a deletion at nucleotide 3107. The NCBI reference sequence (gi:17981852) also shows a deletion at this position when compared with the Anderson sequence (where there is a C) [20]. Since a C at this position is rarer than the 3107 deletion, the deletion was not included when calculating mutational burden. All patients sequenced also had a nucleotide substitution (T to C) at position 1081 in the DLD gene and, as above, this mutation was also not included in the calculation of mutational burden. Mutational burden was compared between RA and OA for each fragment within the *MT-ND1 *amplification region and for the amplified *DLD *region, using a two-tailed Fisher's exact test.

### Known polymorphisms analysis

Reported mtDNA polymorphisms were subtracted from the total and expressed mutational burden and the values were reanalyzed, as above. Published polymorphisms were gathered from Mitomap  and a table of the known polymorphisms found among the patient data is given in Supplementary Table 1.

### Epitope prediction

MHC epitope prediction algorithms were used to search for possible epitope regions within *MT-ND1 *for RA susceptible HLA alleles . The algorithm, MHCPred, used published IC_50 _(median inhibitory concentration) values from radioligand competition assays to develop a predictive algorithm [21]. Given an amino acid sequence, the program predicts the peptides likely to bind to the MHC complex (epitopes) and their IC_50 _values [21]. Peptides with a -logIC_50 _of more than 6.5 are predicted to be binders [21].

## Results

### Mutational burden in OA and RA

**Figure 2:**
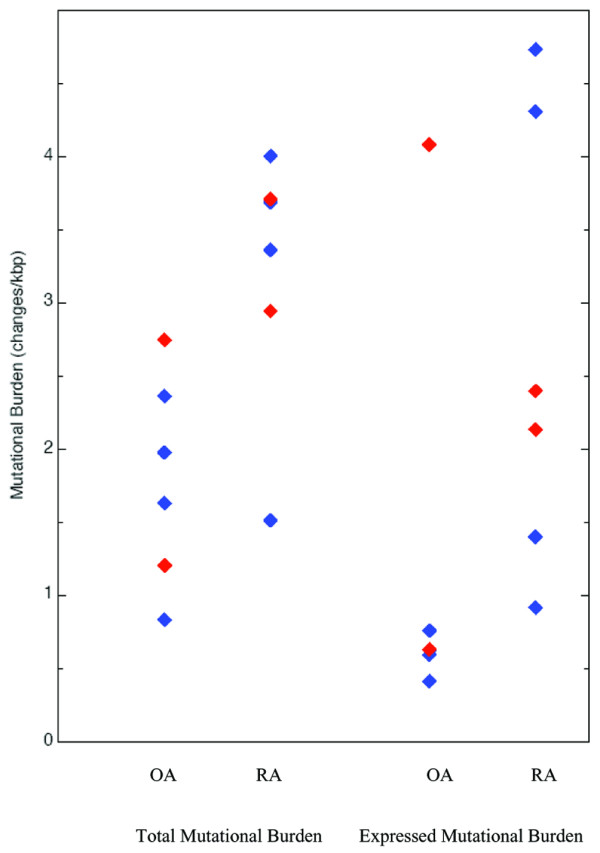
Mitochondrial mutational burden for OA and RA patients. Fibroblast data are given in red, tissue data in blue. kbp, kilobase pairs

OA was used as a nonimmunological-based disease control for the study. We examined both synovial tissue (patients OA227, OA315, OA320, and OA324) and synovial fibroblast lines derived from synovial tissue (patients OA302 and OA304). Approximately 37 kbp were sequenced from OA tissue and 18 kbp from OA fibroblasts, with 67 (2.1/kbp) mutations and 38 (1.8/kbp) mutations found respectively (Table 2; Fig. 2).

For the RA analyses, we also examined both synovial tissue (patients RA301C, RA316, RA317, and RA325) and synovial fibroblast lines (patients RA307 and RA313). Approximately 30 kbp were analyzed from fibroblasts and 18 kbp from tissue, with 101 (3.3/kbp) mutations and 60 (3.4/kbp) mutations found respectively (Table 2; Fig. 2). Comparative analyses of the OA and RA patient data demonstrate significantly more changes per base pair in RA patients than OA (Table 2, Fisher's exact *P *value, ρ < 0.05), whether derived from tissue or fibroblasts. There may be subgroups within the RA or OA set as evidenced by the mutation frequencies between RA and OA patients (Fig. 2). Further studies, with a more detailed patient history, may help correlate mitochondrial mutations to disease factors such as age of onset and response to treatment.

### Amino acid (nonsynonymous) changes

The mutations in the gene for *MT-ND1 *will result in mtND1 protein subunit changes if the mutations created amino acid changes. mtND1 amino acid changes were found in both OA and RA samples. In OA, 7 kbp were analyzed from fibroblast RNA and 16 kbp from tissue RNA. The OA fibroblasts had an expressed mutational burden of 1.7 amino acid changes per kilobase pair (12 changes), and tissue 0.63 amino acid changes per kilobase pair (10 changes) (Table 2; Fig. 2). In RA, 12.4 kbp of the *MT-ND1 *gene were analyzed from fibroblasts and 6.4 kbp from tissue. The RA fibroblasts had an expressed mutational burden of 2.3 amino acid changes per kilobase pair (28 changes), and tissue, 2.5 amino acid changes per kilobase pair (16 changes) (Table 2; Fig. 2).

Thus, there are more amino-acid-changing mutations in RA patients' *MT-ND1 *gene in synovial tissue (*P *< 0.5) (Table 2) than in OA synovial tissue. Although there are more mutations in RA than OA cultured fibroblasts, the expressed mutation frequency is not statistically different (2.5 vs 1.7 amino acid changes per kilobase pair, respectively).

### Nuclear DNA mutational burden

A nuclear gene was analyzed to determine whether it, too, had increased mutations in RA, and thus reveal whether the changes in mutational frequency were specific to mitochondria. The gene, *DLD*, was chosen because its product, dihydrolipoamide dehydrogenase, is a nuclear-encoded mitochondrial subunit peptide, constitutively expressed in all cell types [22]. Mutations were found, as above, in both RA and OA patients. The total mutational burden was high (approximately 2 mutations per kilobase pair); however, there were no significant differences between the RA and OA patient classes (Table 3).

### Epitope prediction and somatic mutations

Several findings suggest that the immune system may aid in the destruction of cells containing mtDNA mutations [11]. Peptides altered by somatic mutations would be presented by MHC and may be recognized as non-self. Searches for possible epitopes in mtND1 led to 76 possible epitopes; of these, 15 were altered by somatic mutations in the RA and OA patients' mitochondrial samples (data not shown). We chose to further analyze the 1B amplified fragment of fibroblasts in more detail because it is totally mRNA-derived (see Fig. 1). We searched all six predicted HLA-DRβ1*0101 epitopes and the ten epitopes with highest -logIC_50 _(*p*IC_50_) values for HLA-DRβ*0401 within the 1B fragment for changes. Changes in epitope regions were noted, and the new mutated epitope was submitted to the MHCPred program for prediction of *p*IC_50 _values (Table 4).

Although RA fibroblasts did not have a statistically higher expressed mutational burden than OA fibroblasts, out of the 16 epitopes investigated, 5 were changed in RA and only 1 was changed in OA. The new (changed) epitopes were analyzed by the same predictive program and all the new RA epitopes fell above the *p*IC_50 _cutoff value of 6.5M while the changed OA epitope fell below this cutoff (Table 4).

## Discussion

These studies revealed that mtDNA somatic mutations were present in the synovium of RA patients at a higher frequency than OA controls. We considered the causes of the somatic mutations (ROS plus selection) as well as the effect these mutations may be having on the etiology and pathogenesis of RA.

### ROS exposure and survival advantage

Exposure to mutagens, such as ROS, can damage both nuclear and mitochondrial DNA. The mtDNA is in close physical proximity to the free-radical-producing process of oxidative phosphorylation and lacks the protective nucleosome structure found in nuclear DNA [23]. Additionally, there is limited ability within mitochondria to repair DNA damage. Together, these attributes make mtDNA highly prone to damage by ROS produced by both mitochondria and exogenous sources [24].

ROS introduces mutations. If the mutations were in genes regulating cell survival, cells that would otherwise stop dividing and die (from DNA damage) may instead proliferate [4]. Insufficient apoptosis of resident synoviocytes and inflammatory cells has been thought to contribute to the persistence of RA [7]. A higher incidence of lymphoma is also well documented in RA, and somatic mutations may lead to enhancement of the aggressive nature of pathogenic cells [25]. For instance, p53 mutations have been found in RA synovial tissues. Their mutations would be predicted to give a growth advantage to the mutated cells, leading to monoclonal expansion [6]. While these mutations are most likely a consequence of inflammation and not the cause of RA, they would be expected to affect disease progression [1].

The NADPH oxidase system of neutrophils and monocytes produces ROS upon activation [26]. Accumulation of these cells within the inflamed joint and the subsequent increase in ROS may be partially responsible for the increased mtDNA mutational burden of RA patients. There are two situations in which nuclear mutations would be expected to occur at greater frequency in RA patients than in OA controls: first, if random processes (ROS from the NADPH oxidase) were the sole cause of the elevated frequency of mtDNA mutations; and second, if the nuclear mutation is conferring an RA-specific characteristic (survival advantage) on the synoviocyte. Examples of the latter instance are the p53 mutations (noted above) that were not found in peripheral blood from RA patients or joint tissue from OA patients. It is thought that p53 is randomly mutated during chronic inflammation by oxygen radicals. Certain mutations within p53 then confer a survival advantage to the synoviocytes, giving them 'transformed' characteristics and participating in the perpetuation of disease [5]. The high frequency of mutations (approximately 2 mutations per kilobase pair) in both patient classes suggests there may be genotoxic stressors in both RA and OA synovia. However, RA synovial tissue and fibroblasts showed no significant increase in the randomly chosen nuclear gene over OA controls. This suggests that the nuclear gene sequenced was not contributing to the progression of RA and that random mutations through exogenous ROS cannot, alone, explain the increase in RA mutational burden found in the mtDNA.

RA is a member of a large class of inflammatory autoimmune diseases. The presence of exogenous ROS produced by neutrophils and monocytes may also be contributing to the pathology of other inflammatory autoimmune diseases. Such a corollary suggests it may be of interest to investigate mtDNA within other inflammatory autoimmune diseases such as systemic lupus erythematosus.

### Altered mitochondrial proteins as non-self

Several findings suggest that the immune system may aid in the destruction of cells containing mtDNA mutations [11]. Peptides altered by somatic mutations would be presented by MHC and may be recognized as non-self. Without a similar analysis of mitochondrial RNA from maternal relatives, it is impossible to say, with certainty, whether all the changes noted were truly somatic mutations. If somatic mutations were changing recognition of mitochondrial peptides from self to non-self, then any inherited changes would be irrelevant. Although we were unable to obtain samples from maternal relatives of patients, there does exist a database of known mitochondrial polymorphisms . When all known polymorphisms were subtracted from the data, the statistical significance of the findings did not change, either for all mutations or just nonsynonymous changes (Table 2).

There are no known data that address the antigenic nature of mitochondrial proteins in RA. However, there is evidence for the involvement of mitochondrial antibodies in another form of arthritis, polymylagia rheumatica. Temporal or giant-cell arteritis is an inflammatory large-vessel disease associated in many patients with polymyalgia rheumatica, and while the etiology of giant-cell arteritis/polymyalgia rheumatica is unclear, there is evidence to support the role of immune mechanisms in its pathogenesis, including the discovery of five autoantigens in patients with the disease [27,28]. Moreover, one of these autoantigens is a mtDNA-encoded subunit of complex IV (cytochrome *c *oxidase subunit II [28]), implicating mtDNA-derived proteins in autoimmune disease.

Our studies predicted numerous regions within mtND1 that may be possible epitopes for HLA-DRβ1*0101 and HLA-DRβ1*0401 (RA-associated HLAs). Five of these possible epitopes were mutated in RA patients and one was mutated in OA. The new (mutated) peptides were analyzed and found to still be possible epitopes for the RA patients, but the OA patient's mutation caused the mutated epitope to fall below the cutoff value for HLA binding (Table 4). Therefore, peptides from mitochondria have the potential to be presented by MHC II, and somatic mutations may alter the peptide such that it is recognized as non-self. As a result, recognition by the immune system of mitochondrial peptides may be aiding in the recruitment of T cells and inflammatory factors, helping to sustain the synovial inflammation characteristic of RA.

## Conclusion

This study demonstrates, for the first time, that mtDNA somatic mutations are present in high frequency in the synovia of RA patients. There are two possible effects of somatic mitochondrial mutations on RA. These somatic mutations may be influencing cellular function, aiding in the acquisition of transformed properties of RA synoviocytes. Second, somatic mutations in peptides displayed by MHC may also be causing an immune reaction, which would further the destructive immune infiltration of the RA joint. The immune system may be primed against these altered peptides because of mimicry with bacterial homologues. Either of these processes would aid in progression of the disease, and earlier immune recognition of mitochondrial peptides may also play a causative role in RA.

## Abbreviations

bp = base pairs; IC_50 _= median inhibitory concentration; MHC = major histocompatibility complex; mtDNA = mitochondrial DNA; NADH = reduced nicotinamide-adenine dinucleotide; NCBI = National Center for Biotechnology Information; OA = osteoarthritis; RA = rheumatoid arthritis; RNS = reactive nitrogen species; ROS = reactive oxygen species.

## Competing interests

The author(s) declare that they have no competing interests.

## Authors' contributions

TD participated in the design of the study, performed some of the molecular genetic studies/analysis, and wrote the manuscript. AC performed the RNA extraction from synovial tissue and established the fibroblast lines. YM participated in the molecular genetic studies and analysis. EK procured the samples and helped in the analysis of data. GW participated in the design of the study, analysis of data, and writing of the manuscript. All authors read and approved the final manuscript.

## Supplementary Material

Additional File 1 A Word file containing a table showing previously published polymorphisms (as of Mar 15, 2005) found within patient samples. Click here for file

## Figures and Tables

**Table 1:** PCR primers and sequence start position for amplification of *MT-ND1 *and *DLD*

Primer name	Start position	Sequence 5'-3'
mt*MT-ND1*^a^		
F1A	2995	TTGGATCAGGACATCCCGA
R1A	3645	ACGGCTAGGCTAGAGGTGG
F1B	3536	TTAGCTCTCACCATCGCT
R1B	4239	ATTGTAATGGGTATGGAGACA
F2	4184	TTCCTACCACTCACCCTAG
R2	4869	CATGTGAGAAGAAGCAG
*DLD*^b^		
DLD – sense	417	ATGATGGAGCAGAAGAGTACTGCA
DLD – antisense	1088	TTTAGTTTGAAATCTGGTATTGAC

^a^See Fig. 1 for position of primers. Both forward and reverse primers in addition to the specific nucleotide sequence have a corresponding M13 tag (M13F, 5'-TGTAAAACGACGGCCAGT- 3' ; M13R, 5'-CAGGAAACAGCTATGACC-3'); start position numbering represents location of 5' end corresponding to the Anderson Reference Sequence [17]. ^b^Start position numbering represents location of the 5' end and corresponds to the DLD cDNA numbering system published by Pons and colleagues [19]. mt, mitochondrial.

**Table 2:** Mitochondrial mutational burden data of OA and RA patient synoviocyte tissue and cultured fibroblasts

		Number of mutations		Total mutational burden (mutations/kbp)		OA vs RA^a^	
		
Mitochondrial mutational burden	Nucleotides sequenced	Initial	Published polymorphisms removed	Initial	Published polymorphisms removed	Initial	Published Polymorphisms removed
Total							
Fibroblasts							
OA	18489	38	20	2.055	1.082		
RA	30503	101	65	3.311	2.131	ρ = 0.01	ρ = 6.9 × 10^-3^
Tissue							
OA	37145	67	40	1.804	1.077		
RA	17663	60	42	3.397	2.378	ρ = 4 × 10^-4^	ρ = 5.1 × 10^-4^
Expressed							
Fibroblasts							
OA	6956	12	7	1.725	1.006		
RA	12394	28	26	2.259	2.098	ρ = 0.5	ρ = 0.10
Tissue							
OA	15805	10	10	0.633	0.633		
RA	6397	16	14	2.501	2.189	ρ = 6 × 10^-4^	ρ = 2.7 × 10^-3^

^a^Two-tailed Fisher's exact test. kbp, kilobase pairs; OA, osteoarthritis; RA, rheumatoid arthritis.

**Table 3:** Nuclear mutational burden data of OA and RA patient tissue and cultured fibroblasts

Patients	Nucleotides sequenced	Number of total mutations	Total mutational burden (mutations/kbp)	Number of amino acid changes	Expressed mutational burden (mutations/kbp)	OA vs RA^a^
Fibroblasts						
OA	10971	36	3.281	24	2.188	
RA	7317	15	2.050	10	1.367	ρ = 0.2
Tissue						
OA	18287	32	1.750	15	0.820	
RA	24522	41	1.672	21	0.856	ρ = 0.2

^a^Two-tailed Fisher's exact test. kbp, kilobase pairs; OA, osteoarthritis; RA, rheumatoid arthritis.

**Table 4:** Predicted epitopes for HLA DRB1*0101 and HLA DRB1*0401 which were changed by nonsynonymous mutations

Patient	Amino acid start position	Predicted core epitope (before mutation)	Predicted -logIC_50 _(M)	New epitope with amino acid change^a^	New predicted -logIC_50 _(M)
HLA DRβ*0101					
RA313	274	RTAYPRFRY	6.661	RTA**H**PRFRY	6.896
RA307	99	NLGLLFILA	6.51	**S**LGLLFILA	6.549
OA302	215	YAAGPFALF	6.681	YAAGPFAL**S**	5.708
HLA DRβ*0401					
RA307	259	FVTKTLLLT	7.329	FV**A**KTLLLT	7.316
RA313	88	PLPMPNPLV	7.093	PLP**I**PNPLV	6.946
RA307	93	NPLVNLNLG	7.09	NPLVNL**S**LG	7.148

^a^Bold indicates amino acid changed by mutation; a -logIC_50 _value above 6.5 is considered to be a binder for prediction purposes. IC_50_, median inhibitory concentration.
