# Supplementary material for: Somatic mutations in the mitochondria of rheumatoid arthritis synoviocytes
Source: Arthritis Res Ther. 2005 Apr 28;7(4):R844–51. doi: 10.1186/ar1752 (PMC1175034; doi:10.1186/ar1752)
Supplement: Additional File 1 — A Word file containing a table showing previously published polymorphisms (as of Mar 15, 2005) found within patient samples. [file ar1752-S1.doc]

Supplemental Table 1: Previously published polymorphisms (as of Mar 15, 2005) found within patient samples. A full table of published polymorphisms can be found at www.mitomap.org.

| **Polymorphism** | **Patient(s)** | **Number of colonies containing polymorphism** | **Reference for polymorphism** |
| --- | --- | --- | --- |
| A3144G | RA317 | 1 | [1] |
| T3350C | OA302 | 1 | [2] |
| T3423G | OA302 | 1 | [3] |
| A3447G | OA320 | 1 | [4], [5] |
| A3480G | OA227, RA307, RA313, RA316 | 2, 6, 2, 1 | [6], [7], [8] |
| G3531A | OA315, RA307 | 3, 3 | [9] |
| C3594T | RA317 | 4 | [10], [11] |
| C3674T | OA302 | 1 | [12] |
| C3738T | RA313 | 1 | [13] |
| T3777C | RA313 | 1 | [14] |
| A3796G | OA304 | 2 | [15], [16] |
| G3834A | RA307 | 1 | [17] |
| T4117C | RA307 | 1 | [18] |
| T4216C | RA313 | 2 | [19] |
| T4471C | RA301C | 2 | [20] |
| T4646C | RA325 | 2 | [7], [21] |
| T4703C | OA302 | 3 | [7], [21] |
| C4763T | OA320 | 1 | [7], [8] |
| A4769G | OA304, OA315, OA320, OA227, OA324, RA301C, RA307, RA313 | 9, 3, 8, 8, 1, 6, 11, 8 | [3] |
| A4833G | OA304 | 1 | [22], [23] |
| C4850T | RA316 | 2 | [13] |

**Works Cited**

1. Brown MD, Voljavec AS, Lott MT, Torroni A, Yang CC, Wallace DC: **Mitochondrial DNA complex I and III mutations associated with Leber's hereditary optic neuropathy**. *Genetics* 1992, **130**(1):163-173.

2. Chalmers RM, Robertson N, Kellar-Wood H, Compston DA, Harding AE: **Sequence of the human homologue of a mitochondrially encoded murine transplantation antigen in patients with multiple sclerosis**. *J Neurol* 1995, **242**(5):332-334.

3. Andrews RM, Kubacka I, Chinnery PF, Lightowlers RN, Turnbull DM, Howell N: **Reanalysis and revision of the Cambridge reference sequence for human mitochondrial DNA**. *Nat Genet* 1999, **23**(2):147.

4. Howell N, Bogolin C, Jamieson R, Marenda DR, Mackey DA: **mtDNA mutations that cause optic neuropathy: how do we know?** *Am J Hum Genet* 1998, **62**(1):196-202.

5. Ikebe S, Tanaka M, Ozawa T: **Point mutations of mitochondrial genome in Parkinson's disease**. *Brain Res Mol Brain Res* 1995, **28**(2):281-295.

6. Thomas AW, Edwards A, Sherratt EJ, Majid A, Gagg J, Alcolado JC: **Molecular scanning of candidate mitochondrial tRNA genes in type 2 (non-insulin dependent) diabetes mellitus**. *J Med Genet* 1996, **33**(3):253-255.

7. Howell N, Kubacka I, Halvorson S, Howell B, McCullough DA, Mackey D: **Phylogenetic analysis of the mitochondrial genomes from Leber hereditary optic neuropathy pedigrees**. *Genetics* 1995, **140**(1):285-302.

8. Howell N, Kubacka I, Halvorson S, Mackey D: **Leber's hereditary optic neuropathy: the etiological role of a mutation in the mitochondrial cytochrome b gene**. *Genetics* 1993, **133**(1):133-136.

9. Abu-Amero KK, Alzahrani AS, Zou M, Shi Y: **High frequency of somatic mitochondrial DNA mutations in human thyroid carcinomas and complex I respiratory defect in thyroid cancer cell lines**. *Oncogene* 2005, **24**(8):1455-1460.

10. Bandelt HJ, Alves-Silva J, Guimaraes PE, Santos MS, Brehm A, Pereira L, Coppa A, Larruga JM, Rengo C, Scozzari R *et al*: **Phylogeography of the human mitochondrial haplogroup L3e: a snapshot of African prehistory and Atlantic slave trade**. *Ann Hum Genet* 2001, **65**(Pt 6):549-563.

11. Chen YS, Torroni A, Excoffier L, Santachiara-Benerecetti AS, Wallace DC: **Analysis of mtDNA variation in African populations reveals the most ancient of all human continent-specific haplogroups**. *Am J Hum Genet* 1995, **57**(1):133-149.

12. Sudoyo H, Suryadi H, Lertrit P, Pramoonjago P, Lyrawati D, Marzuki S: **Asian-specific mtDNA backgrounds associated with the primary G11778A mutation of Leber's hereditary optic neuropathy**. *J Hum Genet* 2002, **47**(11):594-604.

13. Tanaka M, Ozawa T: **Strand asymmetry in human mitochondrial DNA mutations**. *Genomics* 1994, **22**(2):327-335.

14. Ruppert V, Nolte D, Aschenbrenner T, Pankuweit S, Funck R, Maisch B: **Novel point mutations in the mitochondrial DNA detected in patients with dilated cardiomyopathy by screening the whole mitochondrial genome**. *Biochem Biophys Res Commun* 2004, **318**(2):535-543.

15. Pulkes T, Liolitsa D, Nelson IP, Hanna MG: **Classical mitochondrial phenotypes without mtDNA mutations: the possible role of nuclear genes**. *Neurology* 2003, **61**(8):1144-1147.

16. Herrnstadt C, Elson JL, Fahy E, Preston G, Turnbull DM, Anderson C, Ghosh SS, Olefsky JM, Beal MF, Davis RE *et al*: **Reduced-median-network analysis of complete mitochondrial DNA coding-region sequences for the major African, Asian, and European haplogroups**. *Am J Hum Genet* 2002, **70**(5):1152-1171.

17. Liu VW, Shi HH, Cheung AN, Chiu PM, Leung TW, Nagley P, Wong LC, Ngan HY: **High incidence of somatic mitochondrial DNA mutations in human ovarian carcinomas**. *Cancer Res* 2001, **61**(16):5998-6001.

18. Ozawa T, Katsumata K, Hayakawa M, Tanaka M, Sugiyama S, Tanaka T, Itoyama S, Nunoda S, Sekiguchi M: **Genotype and phenotype of severe mitochondrial cardiomyopathy: a recipient of heart transplantation and the genetic control**. *Biochem Biophys Res Commun* 1995, **207**(2):613-620.

19. Rose G, Passarino G, Carrieri G, Altomare K, Greco V, Bertolini S, Bonafe M, Franceschi C, De Benedictis G: **Paradoxes in longevity: sequence analysis of mtDNA haplogroup J in centenarians**. *Eur J Hum Genet* 2001, **9**(9):701-707.

20. Marzuki S, Noer AS, Lertrit P, Thyagarajan D, Kapsa R, Utthanaphol P, Byrne E: **Normal variants of human mitochondrial DNA and translation products: the building of a reference data base**. *Hum Genet* 1991, **88**(2):139-145.

21. Howell N, Kubacka I, Xu M, McCullough DA: **Leber hereditary optic neuropathy: involvement of the mitochondrial ND1 gene and evidence for an intragenic suppressor mutation**. *Am J Hum Genet* 1991, **48**(5):935-942.

22. Ozawa T, Tanaka M, Sugiyama S, Ino H, Ohno K, Hattori K, Ohbayashi T, Ito T, Deguchi H, Kawamura K *et al*: **Patients with idiopathic cardiomyopathy belong to the same mitochondrial DNA gene family of Parkinson's disease and mitochondrial encephalomyopathy**. *Biochem Biophys Res Commun* 1991, **177**(1):518-525.

23. Ozawa T, Tanaka M, Ino H, Ohno K, Sano T, Wada Y, Yoneda M, Tanno Y, Miyatake T, Tanaka T *et al*: **Distinct clustering of point mutations in mitochondrial DNA among patients with mitochondrial encephalomyopathies and with Parkinson's disease**. *Biochem Biophys Res Commun* 1991, **176**(2):938-946.
